# Evaluation of Polybutylene Succinate Composites Reinforced with Lignin and Milled Hemp Stalks

**DOI:** 10.3390/ma19020275

**Published:** 2026-01-09

**Authors:** Nnaemeka Ewurum, Courage Alorbu, Lili Cai, Armando G. McDonald

**Affiliations:** 1 Department of Forest, Rangeland and Fire Sciences, University of Idaho, Moscow, ID 83844, USA; newurum@uidaho.edu (N.E.); calorbu@uidaho.edu (C.A.); 2 Warnell School of Forestry and Natural Resources, University of Georgia, 180 E Green Street, Athens, GA 30602, USA; lcai@uga.edu

**Keywords:** polybutylene-succinate, lignin, hemp-fiber, biocomposites, crosslinking, weathering, biodegradability

## Abstract

This study examines the effects of kraft lignin, milled hemp stalks, and dicumyl peroxide (DCP) crosslinking on polybutylene succinate (PBS) composites, focusing on rheological, mechanical, and thermal properties as well as accelerated weathering and fungal performance. Two composite series were produced via twin-screw extrusion, (a) simple blends (B-series) and (b) DCP-crosslinked formulations (R-series), with emphasis on hybrid lignin–hemp composites (B-PLH and R-PLH). Rheological analysis showed that hemp fiber increased viscosity, while lignin reduced it, and DCP further enhanced shear-thinning behavior. Mechanical testing confirmed that R-PLH exhibited a 16% increase in flexural strength (42.6 MPa) and a 2.4-fold increase in flexural modulus (1785 MPa) over neat PBS, but tensile strength declined by 19%. Thermal analysis revealed a 14–26% reduction in mass loss rate and increased char formation (up to 16.3% in R-PLH), indicating improved thermal stability. Water absorption showed that hemp fibers increased hydrophilicity, further increased by DCP. Accelerated weathering led to significant color change and surface degradation, particularly in R-PLH. Despite lignocellulosic content, all composites exhibited ≤2% fungal degradation, indicating limited mass loss due to fungal exposure under conditions used in this study. Overall, B-PLH and R-PLH offer a balance of stiffness and thermal stability, though trade-offs in tensile strength and weathering resistance should be considered for sustainable applications.

## 1. Introduction

The growing concern created by petroleum-based plastics has led to accelerated interest in biopolymers, especially ones that can be derived sustainably from renewable resources. These materials may be bio-based, biodegradable, or even both, depending on their chemical structure; as a result, they offer several environmental benefits such as lower greenhouse gas emissions and reduced reliance on finite petroleum resources [1,2,3].

Polybutylene succinate (PBS) is a commercially available biodegradable polyester which has been gaining popularity recently. It is produced by reacting succinic acid and 1,4-butanediol. PBS stands out among biodegradable polyesters because of its mechanical versatility and processability. It has a melting point of around 115 °C and a glass transition temperature (T_g_) between −45 °C and −10 °C, which gives it a balance of flexibility and thermal resistance. PBS records tensile strength of 30–35 MPa and elongation at break that exceeds 300%. These are properties that are comparable to conventional plastics like polyethylene-terephthalate (PET) [4,5]. Also, its processability allows for applications in textiles, injection molding, and extrusion. However, PBS has remained limited in large-scale applications primarily because of higher production cost and moderate stiffness compared to conventional plastics like polyethylene (PE) and polypropylene (PP). To address this, recent research has focused on incorporating low-cost bio-based fillers into PBS to improve its stiffness and to reduce raw material cost while maintaining acceptable mechanical performance [4,5,6].

Lignin, which is a major byproduct of the pulping and bioethanol industries, is another bio-based filler of interest. It has an aromatic backbone, thermal properties, and inherent biodegradability, which make it an attractive filler/reinforcement material for biocomposites. When it is blended with PBS, lignin can potentially reduce costs and increase thermal stability [4,5,7]. However, its heterogeneous structure and polarity mismatch with aliphatic polyesters create challenges of interfacial adhesion and uniformity of dispersion. Reactive compatibilization, such as the use of dicumyl peroxide (DCP) to induce crosslinking, has been studied as a strategy for promoting covalent bonding and improved entanglement during melt processing [5,7]. In our earlier work, we demonstrated that kraft lignin can be incorporated into PBS successfully to reduce PBS content while improving stiffness and oxidative stability. However, the addition of lignin alone reduced ductility, melt strength, and caused limited improvement in flexural strength due to insufficient interfacial stress transfer and particle aggregation. This outcome motivated the need to apply a complementary reinforcement that would contribute to mechanical reinforcement while maintaining cost efficiency [5].

Hemp stalk residue is an abundant agricultural byproduct from hemp processing operations. It contains both bast fibers and hurd fractions. The bast fiber fraction has a relatively higher aspect ratio and high crystallinity which promotes stiffness and reinforcement efficiency. The hurd fraction provides lowdensity bulk reinforcement at a low processing cost [8]. Previous studies have shown that incorporating hemp fibers into thermoplastics increases stiffness and melt viscosity, with Young’s modulus values increasing by up to 63% and over 100% in melt viscosity. The use of the mixed hemp residue therefore supports both the enhancement of performance and the valorization of agricultural waste. When combined with lignin, the hemp is expected to contribute to improving mechanical performance, while lignin adds to thermal stability and UV absorption capacity [8,9].

Reactive compatibilization, such as the use of dicumyl peroxide (DCP) to induce crosslinking, has been studied as a strategy for promoting covalent bonding and improved entanglement during melt processing. In PBS-based composites, DCP is expected to improve melt elasticity and filler–matrix bonding without the need for chemical compatibilizers or modifying the surfaces of the fibers. In the composite systems developed in this study, DCP-induced network formation has the potential to offset some of the ductility losses that resulted from filler addition.

Despite the increasing interest in PBS-based biocomposites, the combined effect of hybrid lignin–hemp reinforcement and peroxide-assisted reactive extrusion is not sufficiently understood. In particular, it is not clear how the competing effects of unmodified lignin and hemp fibers would interact with peroxide-induced network modification to simultaneously affect rheological behavior, mechanical performance, thermal stability, and resistance to environmental aging. Accordingly, this study investigates the combined effects of kraft lignin, mixed hemp residue, and peroxide crosslinking on the structural, thermal, mechanical, rheological, as well as the durability behavior of PBS-based composites, with emphasis on evaluating the feasibility of producing cost-reduced, stiffness-enhanced composites for rigid packaging applications compatible with compostable polymer systems where modulus, thermal stability, and material cost are more critical performance criteria than elongation at break. The incorporation of both industrial and agricultural biomass aligns with the current efforts in waste valorization and that of circular material design. It should be noted that the extraction process for lignin affects its chemical structure, and that modifying the filler surfaces and even compatibilization strategies may alter composite performance. However, such effects are beyond the scope of the present study.

Unlike previous studies that developed PBS composites with either natural fibers or lignin individually, this work employs a dual-filler reinforcement strategy that uses kraft lignin and hemp stalk residues while using peroxide-induced crosslinking to promote interfacial interactions through a fully solvent-free melt-processing route. To our knowledge, no prior study has combined these materials and processing conditions while evaluating both performance and environmental durability in the context of rigid packaging applications, which establishes the novelty and relevance of the present study. The filler and peroxide concentrations that were used in this study were selected for a focused evaluation of the structure–property–durability relationship in hybrid PBS–lignin–hemp systems rather than to establish optimized compositions.

Throughout this manuscript, “B-” denotes simple blended formulations without DCP, while “R-” denotes formulations prepared via DCP-assisted reactive extrusion; subscripts indicate filler composition (PL: lignin, PH: hemp fiber, PLH: lignin–hemp).

## 2. Materials and Methods

### 2.1. Materials

This study used PBS pellets (BioPBS™ FZ91PM/FZ91PB, Mitsubishi Chemical Group, Bellevue, OH, USA). The PBS pellets possess a 1.26 g/cm^3^ density and a melt flow rate (MFR) of 5 g/10 min, at 190 °C with a 2.16 kg load. A Thomas–Wiley mill (Swedesboro, NJ, USA) was used to crush the PBS pellets to achieve a particle size of <2 mm, required for blending with lignin and hemp fiber for extrusion. Softwood kraft lignin and milled hemp stalk residues were applied as unmodified bio-based fillers to evaluate baseline composite performance without additional chemical treatments. Dicumyl peroxide (DCP) (99% purity) was used as the crosslinking agent in this study and was procured from Acros Organics (Morris Plains, NJ, USA). The blend of hemp hurd and fiber supplied by Shayne Kimball Farms (Joseph, OR, USA) was processed by milling the hemp fiber in a Wiley mill to pass through a 0.5 mm screen. Softwood kraft lignin (Indulin AT) was supplied by Westvaco (Charleston, SC, USA).

### 2.2. Characterization of Materials

The particle size distribution of Kraft lignin and milled hemp fiber were characterized following a wet-dispersion method on a Bettersizer 2600 (Costa Mesa, CA, USA) particle size analyzer. Gas pycnometry was used to determine the density of kraft lignin and hemp fiber (2 g) using an Ultra-Pycnometer 1000 (Quantachrome, Boynton Beach, FL, USA) with nitrogen as the displacement gas.

### 2.3. Composite Preparation

DCP (1 g) was dissolved in acetone (150 mL) to ensure homogeneity and then thoroughly mixed with milled PBS (400 g) to facilitate uniform dispersal of DCP on the PBS surface. The mixture was dried in a fume hood for 24 h and subsequently in a vacuum oven at 80 °C for 12 h to remove residual solvent. Hemp fiber and kraft lignin were pre-dried at 104 °C for 24 h to eliminate moisture before composite preparation. Two series of composites, comprising simple blends and reactive extruded materials, were prepared using PBS, hemp fiber, and lignin. The simple blends were produced without DCP, while the reactive series contained 0.25% DCP for crosslinking. Various formulations, prepared in 500 g batches, were fed using a weight loss feeder (K-Tron, Sewell, NJ, USA) into an 18 mm co-rotating twin-screw extruder (Leistritz, Branchburg, NJ, USA) with an L/D ratio 40 and operating at 200 rpm. The extrusion temperature profile was set between 120 and 140 °C. The mixtures were extruded into 4 × 50 mm^2^ ribbons. Table 1 summarizes the designations and compositions of the various formulations used in this study.

### 2.4. Gel Fraction

The insoluble gel fraction was assessed for DCP-reacted composites (5 g) by Soxhlet extraction using chloroform (CHCl_3_, 100 mL) as the solvent. The extraction was conducted for 48 h to remove the soluble “sol” fraction. Afterward, the undissolved “gel” fraction was dried in a vacuum oven at 80 °C for 48 h and weighed. The gel fraction yield was calculated using Equation (1):

Gel fraction (%) = (W_gel_/W_0_) × 100 (1)

where W_gel_ is the weight of the dried gel fraction, and W_0_ is the initial weight of the composite sample.

### 2.5. Fourier Transform Infrared Spectroscopy (FTIR)

The FTIR spectra of the composite samples were obtained using a Nicolet iS5 spectrometer (Thermo Scientific, Madison, WI, USA), 64 scans, equipped with an ATR probe and a ZnSe crystal. The Omnic version 9.8 software was used to process the data.

### 2.6. Rheological Behavior

The rheological behavior of PBS, lignin, hemp blends, and DCP crosslinked composites (25 mm Ø × 2.0 mm) was evaluated using a parallel plate rheometer (Bohlin CVO 100 NF, East Brunswick, NJ, USA). Measurements were conducted over a frequency range of 0.01–100 Hz (0.0628–628 rad·s^−1^) at 140 °C, with a strain amplitude of 0.025%. The data were analyzed using Bohlin Rheology version 6.51.0.3 software and Microsoft Excel. Key rheological parameters measured included complex viscosity (η*), elastic modulus (G′), tan δ, and viscous modulus (G″). Shear-thinning behavior was quantified by fitting the rheological data to a modified power-law model, as represented by Equation (2):

|η*(Ꞷ)| = *K*(Ꞷ)^*n* − 1^ (2)

where ∣*η*∗(*ω*)∣ is the complex viscosity as a function of angular frequency ω, *K* is the consistency index, and *n* is the flow behavior index.

### 2.7. Thermal Analysis

Dynamic mechanical analysis (DMA) was performed to determine the viscoelastic properties of rectangular bar specimens (3 mm × 5 mm × 20 mm) which were analyzed in triplicate using a PerkinElmer DMA-7 (Shelton, CT, USA) instrument equipped with a three-point bending fixture (15 mm span). Tests were conducted at 1 Hz frequency and 0.2% strain, over a temperature range of −50 to 120 °C, with a heating rate of 3 °C/min. The data were processed using Pyris v13.3 software. Differential Scanning Calorimetry (DSC) was performed to determine the T_m_, crystallization temperature (T_c_), and degree of crystallinity (*X_c_*) of the extruded composites (10 mg) using a PerkinElmer DSC-7 (Shelton, CT, USA) instrument. The samples were heated to 150 °C at 10 °C/min, cooled to 30 °C at −10 °C/min, and reheated to 150 °C at 10 °C/min. The crystallinity of the polymers (*X*_c_) was calculated using Equation (3).

*X_c_* = Δ*H*_m_/*f*Δ*H*_0_ × 100 (3)

Here, ΔH_m_ refers to the melting enthalpy calculated from the melting endotherm, f is the polymer weight fraction in the formulation, and ΔH_0_ (110.3 J/g) represents the fusion enthalpy of 100% crystalline PBS [10].

Thermogravimetric Analysis (TGA) was performed to assess the thermal stability of hemp fiber, kraft lignin, PBS, and composites (5 mg) using a PerkinElmer TGA-7 (Shelton, CT, USA) instrument. Samples were heated from 30 to 800 °C at a rate of 20 °C/min under nitrogen (30 mL/min). Calibration was achieved using alumel, perkalloy, nickel, and iron standards, and data analysis was performed with Pyris version 13.3.1 software.

### 2.8. Mechanical Properties

Tensile properties of the composites were determined using six replicates of dog-bone specimens (ASTM D638 type 1) [11]. The extruded ribbons were drum-sanded (Grizzly model G0716, Bellingham, WA, USA) to achieve flat surfaces (3 mm thick) and then shaped into dog-bone specimens using a Dremel plunger router fitted with a custom-built jig. Tensile tests were conducted on an Instron 5500R-1132 (Norwood, MA, USA) universal testing machine equipped with Bluehill version 3.3 software. The tests were performed at a crosshead speed of 1 mm/min, with strain measured using an extensometer (model 3542, Epsilon Technology Corp., Jackson, WY, USA). Three-point flexural tests (eight replicates) were performed on machined, extruded specimens (3.2 × 13 × 115 mm^3^) using a 2.5 kN capacity Mecmesin MultiTest-dV (PPT Group, Slinfold, United Kingdom) equipped with VectorPro Lite Software version 6.1.0.0 at a crosshead test speed of 1.1 mm/min according to ASTM standard D790 [12].

### 2.9. Scanning Electron Microscopy (SEM)

The microstructure of the composites’ fractured surfaces was analyzed using scanning electron microscopy (SEM). The samples were gold-coated prior to imaging using a Zeiss Supra 55 VP-FEG (Dublin, CA, USA) equipped with an SE2 detector. Imaging was performed at an accelerating voltage of 10 kV.

### 2.10. Water Absorption

Water absorption tests were performed on composite specimens (18 mm × 18 mm × 3 mm) in triplicate. Samples were immersed in a water bath at room temperature for 56 d. Gravimetric measurements were taken daily during the first week and weekly for the remainder of the test period to monitor weight gain. The diffusivity (D_f_) was calculated using Equation (4).

(4) $$D_{f}=\pi {\left(\frac{h}{{4M}_{f}}\right)}^{2}{\left(\frac{\delta MC}{\delta \surd t}\right)}^{2}$$

where M_f_ is Max moisture content at the end, h is sample thickness in meters, and M/√t is the initial slope from the plot MC vs. √t.

### 2.11. Accelerated Weathering of PBS and Composites

Accelerated weathering tests were conducted on composite samples (in triplicate) using a xenon-arc weatherometer (Q-Sun Xe-1-S, Westlake, OH, USA). The samples were subjected to a cyclic exposure regime consisting of 2 h of radiation followed by 2 h of radiation combined with water spray. The irradiance was maintained at 0.70 W/m^2^ at 340 nm, and the chamber temperature was approximately 70 °C during the radiation step. Samples were collected weekly for analysis [13]. Color measurements (FRU model WR-10QC, Longgang, Shenzhen, China) were taken at three different locations on each sample, with three replicates per WPC formulation. Color was expressed using the CIELAB system, which consists of L* (lightness), a* (red–green chromaticity), and b* (yellow–blue chromaticity) coordinates based on a D65 light source. The total color change (ΔE_ab_) was calculated using the Euclidean distance formula:

ΔE_ab_ = √(ΔL^2^ + Δa^2^ + Δb^2^) (5)

where ΔL, Δa, and Δb represent the differences between the initial and final values of L*, a*, and b*, respectively. An increase in the L* value indicates lightening of the sample. A positive Δa indicates a shift towards red and a negative Δa indicates a shift towards green. The b* coordinate represents the yellow–blue axis, where a positive Δb indicates a shift towards yellow and a negative Δb indicates a shift towards blue.

To quantify the changes in surface chemistry due to UV-induced degradation, key functional group indices were calculated using FTIR absorbance ratios, lignin (LI, 1515–1508 cm^−1^), hydroxyl (HI, 3080–3500 cm^−1^), carbonyl (CI, 1650–1850 cm^−1^), and wood (WI, 1100–1200 cm^−1^), each determined as a ratio of the absorbance within the band of interest to the absorbance of the C–H stretching band (2800–3000 cm^−1^) which serves as an internal reference.

### 2.12. Biodegradation of Composites

A fungal durability test was conducted using the soil-block test method following the American Wood Protection Association (AWPA) Standard E10-16 [14] with minor modifications in the sterilization of samples [15]. Twelve extruded WPC block specimens (3 × 20 × 20 mm^3^) of each treatment group were dried in a vacuum oven at 80 °C, weighed (*m_i_*), soaked in distilled water for 24 h, and sterilized by dipping samples in 70% ethanol solution in water and allowed to dry in the fume hood for 1.5 h. Six replicates of composite samples from each treatment group were exposed to actively growing culture bottles of a brown rot fungus, *Gloeophyllum trabeum* (GT), and a white rot fungus, *Trametes versicolor* (TV), for 16 weeks. Six replicate sapwood samples of loblolly pine (*Pinus taeda* L.) and poplar (*Liriodendron tulipifera* L.) (5 mm × 18 mm × 18 mm) were exposed to GT and TV fungi, respectively, for 4 weeks to confirm the activeness of the decay culture bottles during the exposure period. At the end of the incubation period, the composite specimens were removed, cleaned of adhering mycelium, and weighed to determine their wet weight. The samples were oven-dried and reweighed (*m_d_*) to calculate mass loss due to decay as follows:

(6) Mass loss %=((mi −md)/mi) × 100%

where *m_i_* and *m_d_* are the mass of vacuum oven-dried (80 °C) wood samples before and after fungal exposure, respectively. FTIR spectra of the fungi-exposed samples were collected and processed following the same protocols described in Section 2.5 and Section 2.11. This fungal test was used to assess biological durability under laboratory decay conditions and does not represent a standard compostability test.

### 2.13. Data Analysis

Statistical analyses were performed using Tukey’s pair-wise comparisons and *t*-tests to assess differences among formulations. Results were deemed statistically significant at a 95% confidence level (*p* < 0.05).

## 3. Results and Discussion

### 3.1. Extrusion of PBS–Lignin–Hemp Formulations

To achieve homogeneous mixing, PBS was milled into granular powder and subsequently blended with kraft lignin powder (average particle size of 166 µm) and/or milled hemp fiber (average particle size of 232 µm). Two series of formulations were prepared by twin-screw compounding/extrusion—simple blends of PBS–lignin–hemp fiber and DCP crosslinked composites. The gel fraction is an indication of the insoluble fraction formed during reactive extrusion in the R-series (reactively extruded with DCP). The simple blends will be soluble in CHCl_3_, while the DCP crosslinked formulations will form a three-dimensional network gel. The gel fraction results (Table 2) show that R-PBS showed a high apparent gel fraction (93.8%), which likely reflects limited solubility and the presence of insoluble or crystalline fractions following reactive extrusion; however, the inclusion of lignin for R-PL significantly hindered crosslinking, leading to a gel fraction of only 4.3% due to the inherent incompatibility between PBS and unmodified lignin [5,16,17]. R-PH recorded a gel content of 69.6%, suggesting effective DCP-induced crosslinking of the PBS matrix in the presence of hemp fibers. R-PLH showed an intermediate gel content of 41.2%, which points to the opposing effects of hemp and lignin. The density measurements are summarized in Table 2. Hemp fibers, lignin, and B-PBS recorded density values of 1.42, 1.21, and 1.24 g/cm^3^, respectively, while the addition of lignin (B-PL, 1.29 g/cm^3^) and hemp fibers (B-PH, 1.31 g/cm^3^) to B-PBS increased the density. The combined lignin–hemp fiber composites (B-PLH, 1.31 g/cm^3^) showed a comparable density to B-PH, which suggests that hemp fibers contribute more to the overall mass and structural integrity of the composite materials compared to lignin [18]. The reacted composites had density values comparable to their blended counterparts.

### 3.2. FTIR of PBS and PBS–Lignin–Hemp Composites

**Figure 1:**
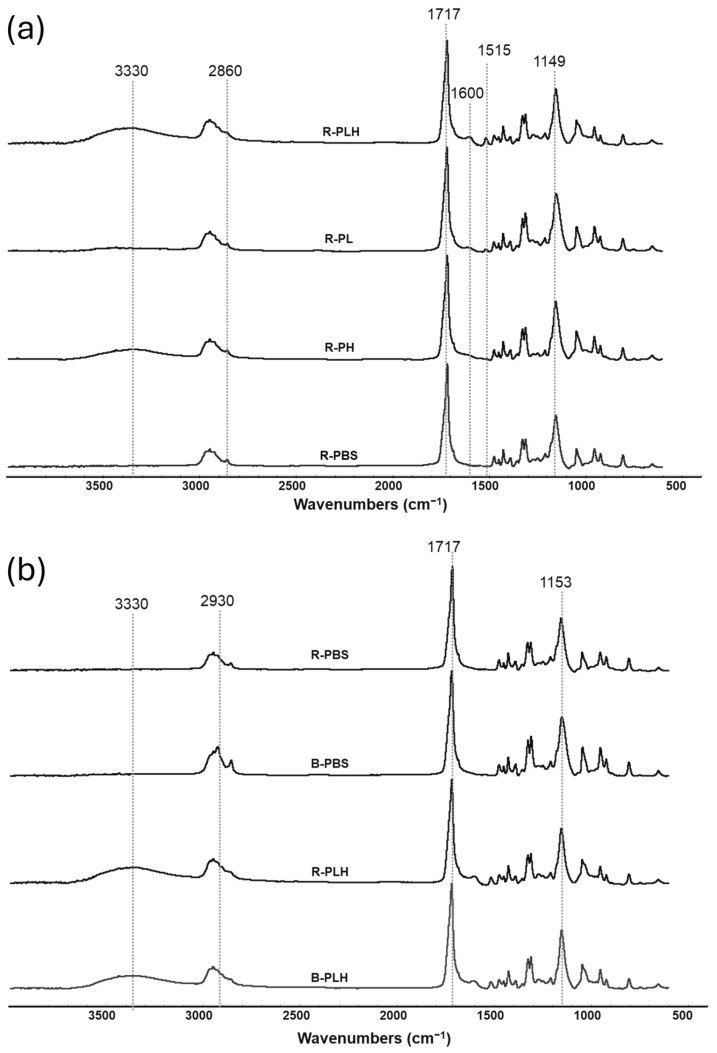
FTIR spectra of (**a**) DCP crosslinked PBS–lignin–hemp composites and (**b**) blended PBS–lignin–hemp composites.

The chemical functional groups of the PBS composites were investigated by FTIR. Figure 1a shows the FTIR spectra of the R-series composites (R-PBS, R-PL, R-PH, and R-PLH), focusing on the chemical changes from adding lignin and hemp fibers to the PBS matrix. In the O–H stretching region (~3400–3500 cm^−1^), R-PBS had a barely detectable band, typical of plastics with minimal hydroxyl content. The intensity increased slightly for R-PL due to lignin’s hydroxyl groups and significantly for R-PH, reflecting the cellulose-rich hemp fibers. R-PLH showed the visually strongest aromatic and hydroxyl-associated features, reflecting its combined lignin–hemp composition [19,20]. In the C–H stretching region (~2850–3000 cm^−1^), R-PBS showed a sharp but low-intensity band at 2860 cm^−1^. The intensity increased for R-PL and R-PH, with R-PLH showing the strongest C–H bands, attributed to cellulose and hemicellulose from hemp fibers. The peak broadening observed in R-PH and R-PLH suggests increased compositional heterogeneity that is associated with lignin and hemp fiber incorporation, although FTIR alone is not sufficient to confirm matrix–filler interaction. The carbonyl stretching region (~1710–1740 cm^−1^) showed a major band at 1714 cm^−1^ for R-PBS, characteristic of PBS ester bonds. The aromatic region (~1600 cm^−1^) was flat for R-PBS but increased in intensity for R-PL and R-PH, with the highest intensity for R-PLH due to contributions from lignin’s aromatic structure and hemp fibers. Similarly, the band at 1515 cm^−1^, attributed to lignin’s aromatic skeletal vibrations, was most pronounced in R-PL and R-PLH. At 1152 cm^−1^, all samples showed similar bands, except R-PL, which showed a variation near 1149 cm^−1^; the slight shift reflects normal compositional differences but does not indicate network disruption [21].

Figure 1b compares the spectra of reacted (R-PBS, R-PLH) and blended (B-PBS, B-PLH) formulations to assess the spectral difference associated with DCP. In neat PBS (B-PBS vs. R-PBS), small differences were observed at the C–H stretching (~2800–3000 cm^−1^), carbonyl (1714–1717 cm^−1^), and the C–O stretching (1150 cm^−1^ to 1153 cm^−1^) regions.

In lignin–hemp composites (B-PLH vs. R-PLH), the O–H and C=O regions appeared more pronounced after DCP treatment, although FTIR alone cannot determine whether these changes are from oxidation, hydrophilicity or other processing effects. Although DCP may influence composite structure, FTIR did not show clear spectral evidence to confirm such changes.

### 3.3. Rheology of PBS and PBS–Lignin–Hemp Composites

**Figure 2:**
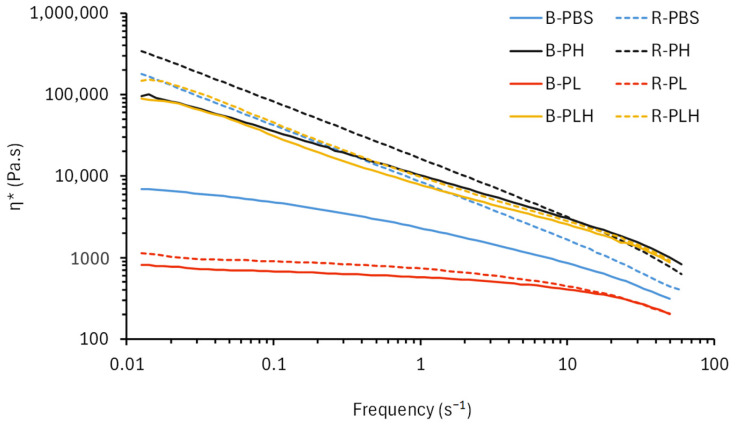
Flow curves (η* versus frequency) of blended and crosslinked PBS–lignin–hemp composites.

The flow characteristics (complex viscosity (*η*∗) versus frequency) of PBS, PBS–lignin–hemp fiber blends, and reacted PBS–lignin–hemp fiber were characterized under dynamic conditions at 140 °C; the results are shown in Figure 2. A non-Newtonian, shear-thinning response was observed for the polymer melts. Therefore, each formulation data was fitted to a power-law model (Equation (2)), and a summary of the model parameters (*K*, *n* and R^2^) are given in Table 3. All fitted models produced coefficients of determination (R^2^) greater than 0.9. DCP crosslinking increased the viscosity of PBS, as seen when comparing B-PBS and R-PBS, and observed by Feng et al. (2016) [22]. For comparative purposes between samples, the η* at 1 Hz was used and is discussed below. The *η*∗ increased from 2.24 kPa·s in B-PBS to 8.15 kPa·s in R-PBS. This increase is consistent with restricted chain mobility and improved network connectivity that is associated with peroxide-induced modification. The *n* value decreased from 0.632 in B-PBS to 0.284 in R-PBS, which indicates stronger shear-thinning behavior in the crosslinked matrix, consistent with reports of peroxide-induced melt-strength improvements in PBS systems [7,22]. Similar trends were observed in R-PH, where DCP further increased *η*∗ and decreased *n* compared to B-PH [23]. B-PL had the weakest shear-thinning behavior (*n* = 0.875) and the lowest *η*∗ value of 0.573 kPa·s. This shows that lignin acted as a plasticizer to PBS [24,25]. DCP reaction slightly improved *η*∗ in R-PL by 27% as compared to B-PBS. The addition of hemp fibers to PBS (B-PH) contributed more significantly to the polymer melt *η*∗ (4-fold increase), and DCP crosslinking (R-PH) further increased *η*∗ to 15.7 kPa·s at 1 Hz. R-PH had the lowest *n* (0.276), indicating enhanced shear-thinning behavior, which is consistent with increased melt elasticity in the presence of hemp fibers and DCP [7,23]. An interactive effect was observed when lignin and hemp fibers were combined, particularly in the reacted system. While B-PLH recorded moderate improvements (7.57 kPa·s), R-PLH showed a further increase in viscosity by 24%. The *n* values for all DCP-reacted samples were lower than the corresponding blended samples and support the role of DCP in promoting compatibility between PBS, lignin, and hemp fibers [7,23].

### 3.4. Dynamic Mechanical Analysis (DMA)

Viscoelastic properties (E′ and tan δ) of the various PBS–lignin formulations were determined by DMA to examine the influence of filler/reinforcement content and crosslinking (Table 4). At −48 °C, R-PBS recorded a higher *E*′ (1.14 GPa) than B-PBS (0.98 GPa), which suggests increased stiffness due to DCP crosslinking. The *E*′ values at −48 °C showed that the addition of lignin alone (B-PL and R-PL) reduced stiffness compared to neat PBS. This decrease is likely due to lignin’s heterogeneous and amorphous nature, which can disrupt the uniformity of the PBS matrix and reduce its ability to store elastic energy [26]. A study by Young et al. supports this observation, indicating that copolymers of lignin with various polymers consistently recorded lower E′ values compared to their neat counterparts [27]. In contrast, the addition of hemp fibers increased the modulus, with B-PH (1.01 GPa) and B-PLH (1.51 GPa) outperforming B-PBS. The addition of DCP further improved stiffness, as seen in R-PH (1.21 GPa) and R-PLH (1.68 GPa), which confirms improved matrix–filler interactions and reduced mobility in the crosslinked series [28]. At 10 °C, all the formulations showed a decrease in *E*′, which is consistent with the thermal softening of the polymer matrix. However, R-PLH maintained the highest modulus (0.81 GPa). The tan δ values at −48 °C and 10 °C showed that the formulations with lignin and hemp fibers generally had lower damping behavior compared to the neat PBS; this further suggests that the addition of these natural fibers contributed to a more rigid and stable structure [29,30]. The T_g_ was determined from tan δ max values (Table 4). The T_g_ of PBS increased slightly with the addition of fillers and DCP crosslinking. Neat PBS formulations (B-PBS and R-PBS) had T_g_ values of −34 °C and −33 °C, respectively. The addition of lignin (B-PL and R-PL) increased T_g_ to −27 °C and −26 °C, respectively, which is most likely due to the inherently high T_g_ of kraft lignin (147 °C) [31]. Hemp fibers, on the other hand, (B-PH and R-PH) slightly increased T_g_ to −33 °C and −29 °C. The combined lignin–hemp composites (B-PLH and R-PLH) maintained T_g_ at −26 °C and −27 °C, respectively, which reflects a balance between filler contributions and crosslinking effects.

### 3.5. Differential Scanning Calorimetry

**Figure 3:**
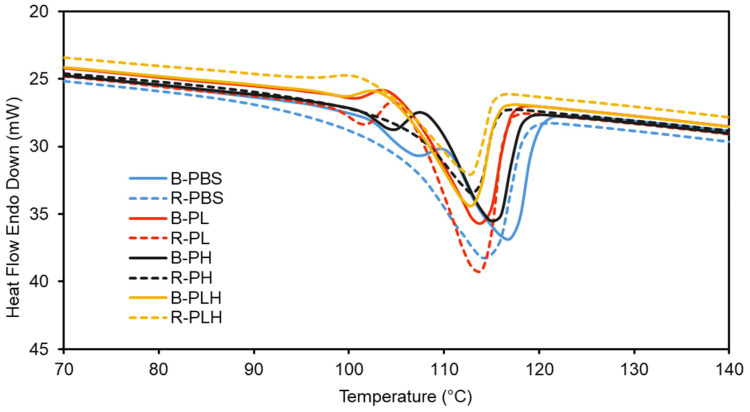
DSC thermograms (2nd heating cycle) of blended and reacted PBS and PBS–lignin–hemp composites.

The thermal properties (X_c_, T_c_, and T_m_) of PBS–lignin–hemp fiber formulations were determined by DSC (Figure 3 and Table 5). The DSC thermograms (Figure 3) show that all samples, except for R-PBS, R-PH, and R-PLH, showed a small initial peak T_m1_ around 102–108 °C before the main melting peak, T_m2_ at 112–116 °C. These initial peaks may indicate the melting of less stable crystalline regions within the polymer matrix [32,33]. In the reacted composites (R-PBS, R-PH, and R-PLH), the absence of the initial melt peaks indicates that DCP crosslinking reorganized the polymer matrix, eliminating these imperfections and producing a more uniform crystalline phase [34]. In addition, The DSC thermograms of R-PLH showed a cold crystallization peak (T_cc_) around 101 °C before the primary T_m_ at 113 °C, a feature absent in the non-crosslinked B-PLH. This cold crystallization suggests that crosslinking via DCP introduces structural constraints within the PBS matrix, which promotes the reorganization of polymer chains during heating. T_m2_ values for the formulations were relatively consistent, with B-PBS, R-PBS, and B-PH showing T_m2_ values around 115–116 °C. In comparison, the formulations containing lignin (B-PL and R-PL) had a slightly lower T_m2_ value of 114 °C. This suggests that crosslinking and the presence of fillers/reinforcement only had minor disruptive effects on the crystalline phases of PBS [35,36]. T_c_ showed more variability, with R-PBS, R-PL, and R-PH recording higher T_c_ values (92.7 °C, 80.0 °C, and 93.9 °C, respectively) than their blended counterparts. This increase in T_c_ for the reacted formulations may be attributed to the crosslinking effects of DCP, which improved the nucleating effect of fillers and created a more organized crystalline structure [37]. *X_c_* increased with DCP crosslinking, from 25.0% in B-PBS to 29.1% in R-PBS and similarly for B-PH to R-PH. R-PLH also showed higher *X_c_* (30.6%) compared to B-PLH (28.6%), indicating that DCP improved filler dispersion and enhanced crystalline ordering [38].

### 3.6. Thermogravimetric Analysis

The thermal degradation behaviors of lignin, hemp fiber, PBS, and various PBS–lignin–hemp fiber blended/reacted formulations were determined by TGA and differential thermogravimetric (DTG) analysis (Table 6). The thermal degradation onset (T_onset_) of lignin and hemp fiber started at 320 °C and 315 °C, respectively. The T_onset_ for B-PBS was 393 °C (B-PBS) and for R-PBS was 392 °C (Table 6). PBS formulations containing lignin or hemp fibers (B-PL, R-PL, B-PH, and R-PH) recorded lower T_onset_ values in the range of 380–388 °C, which can be attributed to the presence of lignin and hemp fibers. Lignin and hemp fibers introduced additional thermal degradation pathways, which in turn resulted in the earlier onset of degradation [39]. The combined effect of lignin and hemp fiber (B-PLH and R-PLH) caused a further 20 °C decrease in T_onset_. From the DTG curves, the temperature at the peak maximum (DTG_max_) were determined. The DTG_max_ for B-PBS and R-PBS are recorded at 433 °C and 435 °C. The formulations containing lignin and/or hemp fibers showed lower DTG_max_ values ranging from 418 to 425 °C, again suggesting that the presence of lignin and hemp fibers may lead to a reduction in the thermal stability of the composites [39,40]. However, the intensity of the DTG_max_ peaks, which corresponds to the %mass loss rates of the materials, show that the addition of lignin/hemp fibers caused a 14–26% decrease in mass loss rate compared to B-PBS (−42%/min) and R-PBS (−41%/min). The combined lignin–hemp fiber composites recorded even more pronounced improvements in mass loss rates, 50% for B-PLH and 48% for R-PLH. A similar trend is observed with the residual mass at 900 °C, with B-PL and R-PL showing significantly higher residual percentages (around 101%) compared to B-PBS and R-PBS (1.4–1.6%). The combination of lignin and hemp fibers increased residual mass even further in B-PLH (12%) and R-PLH (16.3%), indicating that lignin and hemp fiber contributed to the formation of char during thermal degradation, which can enhance the material’s thermal stability and fire resistance properties [39,40]. DCP crosslinking was not found to significantly impact thermal degradation properties of PBS and PBS-based composites.

### 3.7. Mechanical Properties of PBS and PBS-Based Biocomposites

The tensile properties of extruded PBS and PBS–lignin–hemp formulations were determined, and the results are given in Table 7. Neat PBS (B-PBs and R-PBS) recorded the highest tensile strength values of around 36.7 MPa, consistent with the literature [41]. DCP crosslinking of PBS did not change its tensile strength. The addition of fillers reduced tensile strength compared to neat PBS, indicating that, while providing some level of reinforcement, the presence of fillers in PBS may not achieve the same level of stress transfer as the PBS matrix alone [41,42,43]. R-PH showed improved tensile strength (32.1 MPa) over B-PH (26.5 MPa), indicating that DCP crosslinking enhanced the performance of PBS–hemp composites. Lignin reduced the tensile strength of PBS due to plasticization.

Tensile elongation at break (EAB) decreased significantly (*p* < 0.05) with the incorporation of lignin and hemp. Neat PBS (12.4–15.6%) recorded the highest ductility while the lignin–hemp composites showed the least EAB (2.4–2.6%). The presence of the fillers limited the polymer chain mobility, making the material less ductile. Crosslinking did not restore ductility.

The trend in modulus agrees with this interpretation. The Young’s modulus for B-PBS was 738 MPa and increased by 18% due to DCP crosslinking. The Young’s moduli progressively increased with the addition of lignin (928–967 MPa), hemp fiber (1490–1540 MPa), and lignin plus hemp fiber (1869–1895 MPa). The increase in stiffness aligns with the literature describing lignocellulosic rigid reinforcement in PBS composites [41,42,43], while crosslinking had a comparatively minimal effect on the modulus within the same formulation types.

**Figure 4:**
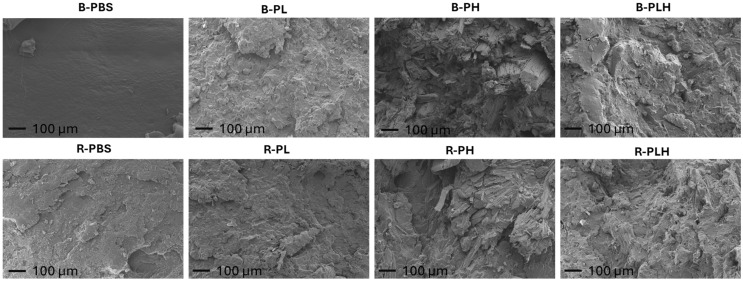
SEM micrographs showing fracture surfaces of PBS, PBS–lignin, PBS–hemp and PBS–hemp–lignin composites at 100× magnification.

SEM analysis was performed on tensile fracture surfaces to evaluate failure characteristics (Figure 4). B-PBS had a smooth, homogeneous surface indicative of ductile failure with minimal obstacles to crack propagation [44].

R-PBS showed slightly rougher topography which may be associated with differences in fracture morphology. B-PL and R-PL showed similar rough, irregular surfaces with visible voids, indicating weak matrix–filler bonding and phase separation [25,45]. There is no clear improvement in surface morphology with DCP reaction in these PBS–lignin composites. B-PH had a rough, fibrous surface with noticeable fiber pull-outs and gaps around fibers, suggesting poor fiber–matrix adhesion [4]. R-PH showed fewer fiber pull-outs and more fractured fibers, indicating improved bonding after crosslinking. The fracture surfaces of B-PLH and R-PLH are smoother and more cohesive compared to B-PH, with fewer gaps and less fiber pull-out, suggesting that lignin improved surface morphology by filling voids. However, there were no apparent morphological differences observed between B-PLH and R-PLH at the SEM scale. Given the qualitative nature of SEM, molecular-level modifications may not be readily distinguishable.

The flexural properties of PBS and PBS–lignin–hemp formulations showed a similar trend and are summarized in Table 7. Neat PBS formulations showed the lowest values, with B-PBS and R-PBS exhibiting flexural strengths (FS) of 29.0 MPa and 30.5 MPa, respectively, and flexural moduli (FM) of 701 MPa and 759 MPa, indicating minimal improvement from crosslinking. The addition of fillers significantly enhanced both properties compared to neat PBS, as indicated by different subscript letter groupings in Table 7 (*p* < 0.05). This is consistent with studies which have found lignin [41] and hemp [8] to improve the flexural properties of thermoplastics. Hemp-based composites showed particularly pronounced increases, with B-PH achieving a FS of 31.8 MPa and FM of 1071 MPa, while R-PH exhibited substantial improvements to 45.5 MPa (FS) and 1370 MPa (FM), corresponding to a change from group “cd/d” to group “a”, reflecting the reinforcing contribution of hemp and statistically significant changes associated with DCP-assisted reactive extrusion. Lignin-based composites also improved, with B-PL having a FS of 32.1 MPa and FM of 830 MPa and R-PL showing increases to FS of 35.8 MPa and FM of 930 MPa, highlighting the stiffening effect of lignin and the contribution of crosslinking [46,47]. The highest values were observed in lignin–hemp composites, with B-PLH having a FS of 37.6 MPa and FM of 1496 MPa and R-PLH achieving a FS of 42.6 MPa and FM of 1785 MPa, demonstrating the synergistic effect of both fillers and the significant enhancement from DCP crosslinking.

### 3.8. Water Absorption Testing of Composites

The WA behavior (1 d and 56 d) of the PBS-based composites varied significantly based on the type of filler and the presence of DCP crosslinking (Table 8). B-PBS and R-PBS had the lowest water uptake (~1% at 56 d). The addition of lignin (B-PL, R-PL) increased water absorption (2.9–3.3%), albeit modestly, as lignin contains hydrophilic hydroxyl groups yet retains some water-repellent characteristics due to its aromatic structure [35]. Similar water absorption behavior has been reported for PBS–lignin composites with WA values of 3.54% and 3.77% for unmodified and modified lignin composites [35]. Hemp-filled composites (B-PH, R-PH) showed significantly higher water absorption (3.6–4.4%), owing to the hydrophilic nature of lignocellulosic fiber [48]. Kenaf core fiber composites had higher WA of 5.7% [35]. The highest WA was recorded in the PBS–lignin–hemp composites (B-PLH, R-PLH), with R-PLH absorbing 8.3% water, reflecting the combined hydrophilic nature of both fillers. The presence of DCP led to a significant increase in WA in the R-PLH and R-PL composites. This could be attributable to polar groups generated via oxidation by the free radical initiator [49]. Diffusion coefficients were determined from the WA time plots, and for B-PBS a value of 6.81 × 10^−12^ m^2^/s was obtained. The diffusion coefficient increased with the addition of hydrophilic fillers [50]. TS increased significantly with the addition of hemp fibers, while lignin alone had little impact, with B-PL and R-PL swelling <0.70% at 56 d. B-PBS and R-PBS showed the lowest TS of about 0.66%. The PBS–lignin–hemp composites showed the highest swelling at about 3.5%.

### 3.9. Accelerated Weathering Performance of PBS and PBS–Lignin–Hemp Composites

#### 3.9.1. Color Change

**Figure 5:**
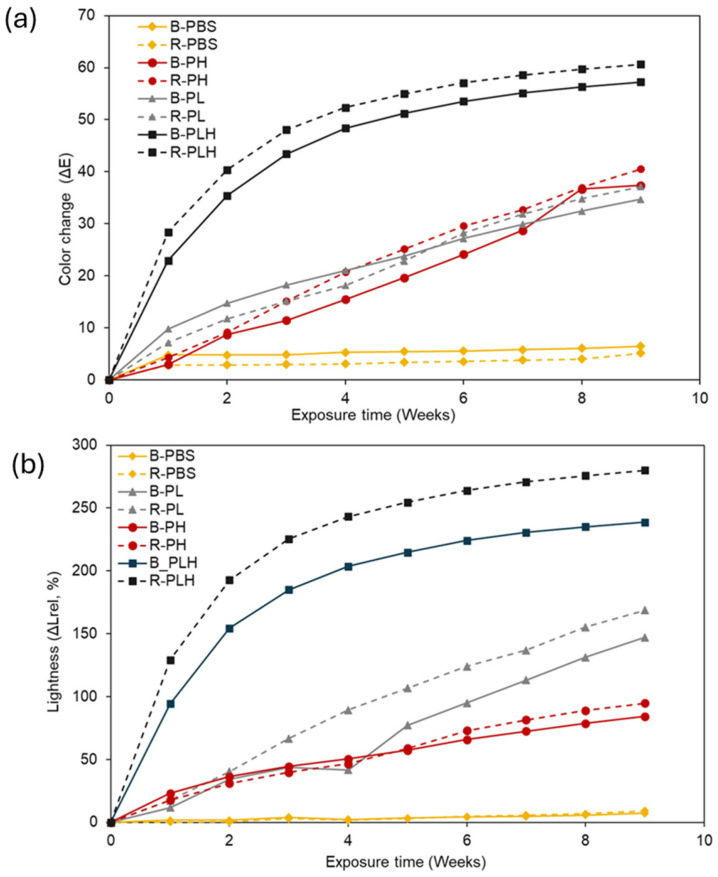
Effect of UVA accelerated weathering exposure time on the (**a**) color change (ΔEab) and (**b**) lightness (ΔL) of PBS–lignin–hemp composite.

The color stability of PBS and PBS-based composites was assessed by monitoring changes in relative lightness (Δ*L*_rel_) and total color change (ΔE_ab_) over a 9-week accelerated weathering period (Figure 5). B-PBS and R-PBS exhibited minimal color change, with Δ*L*_rel_ below 10% and Δ*E_ab_* around 10 units, indicating inherent UV–radiation resistance due to the absence of lignocellulosic fillers. In contrast, composites with lignin, hemp fibers, or both showed significantly higher Δ*L*_rel_ and Δ*E_ab_* values, reflecting greater UV-induced degradation. Lignin contains chromophoric groups that degrade under UV light, causing fading and color shifts [51]. B-PH and R-PH showed higher color changes compared to B-PL and R-PL samples. The crosslinked R-PH showed greater Δ*L*_rel_ and Δ*E_ab_* values than B-PH. This suggests that DCP crosslinking introduced polar groups through oxidation, making the composites more prone to water absorption and UV-induced degradation. In addition, hydrophilic hemp fibers can absorb water and swell the composite, then dry and shrink, thus leading to cracks and degradation under UV exposure [52]. The PBS–lignin–hemp composites (B-PLH and R-PLH) experienced the most severe color changes (Δ*E_ab_* > 60 units.), with Δ*L*_rel_ reaching 239% for B-PLH and 280% for R-PLH. The composites changed from dark brown to a chalky white color after weathering and were similar to that observed by other studies [52,53,54]. The higher filler content in these hybrids likely contributed to their greater color instability [53]. Lignin-only composites (B-PL and R-PL) showed moderate color changes (Δ*E_ab_* ~ 37 units), with Δ*L_rel_* reaching 147% and 169%, respectively. The UV-absorbing properties of lignin can slow down matrix degradation; however, photooxidation of lignin itself leads to moderate color changes over time [55].

#### 3.9.2. Surface Morphology of Weathered Materials

**Figure 6:**
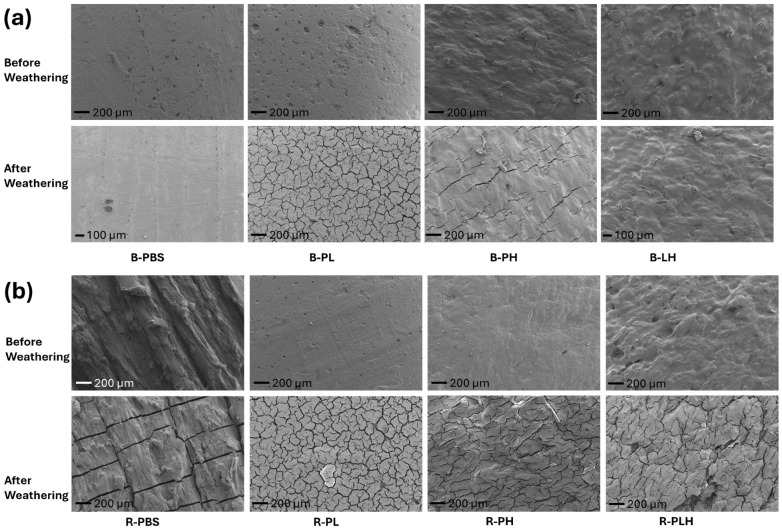
Scanning electron micrographs showing the effects of weathering on (**a**) blended and (**b**) DCP-reacted PBS–lignin–hemp composite.

The surface morphology of non-weathered and 9-week weathered PBS and PBS composites by SEM are shown in Figure 6. The SEM micrographs of B-PBS showed better resistance to weathering (no cracks) compared to R-PBS (Figure 6). Severe surface cracks were seen in all DCP-crosslinked composite (R-PBS, R-PL, R-PH, and R-PLH) samples after weathering, while the B-PL and B-PH blended samples also showed cracks. The B-PLH composites showed no cracking, and the combination of lignin and hemp fibers appeared to reduce crack propagation. Moisture and UV exposure of natural fibers are known to cause photodegradation, leading to fiber pull-out and surface roughness [52,54]. B-PH had more visible cracks, likely due to stress concentrations from hemp fibers, while B-PL showed the most extensive cracking, consistent with the susceptibility of lignin to UV-induced photooxidation [54,56]. The DCP-induced crosslinked samples were more oxidized, and this introduces polar groups, increasing the material’s affinity for moisture and oxygen [57]. In addition, the DCP-reacted samples had a higher crystallinity (by DSC) than the blended samples, making the material more rigid and brittle, thus making the material more prone to cracking under environmental stress [54,58]. The R-PLH material showed the most severe cracking among all samples, suggesting that the combination of lignin, hemp fibers, and DCP crosslinking made the composite particularly susceptible to UV-induced damage [53,54]. The trends show performance trade-offs that were introduced by lignocellulosic reinforcement and DCP-assisted reactive extrusion. Although crosslinking and filler addition improved stiffness, thermal stability, and flexural performance, they also restricted polymer chain mobility which resulted in reduced tensile strength and elongation at break. The DCP-reacted samples showed more pronounced surface cracking and discoloration under accelerated UV exposure, which is attributed to increased surface oxidation and embrittlement in the crosslinked materials. These results indicate that the improvements in stiffness and thermal stability may come at the expense of increased susceptibility to photo-induced degradation.

#### 3.9.3. Surface Chemistry of Weathered Samples

**Figure 7:**
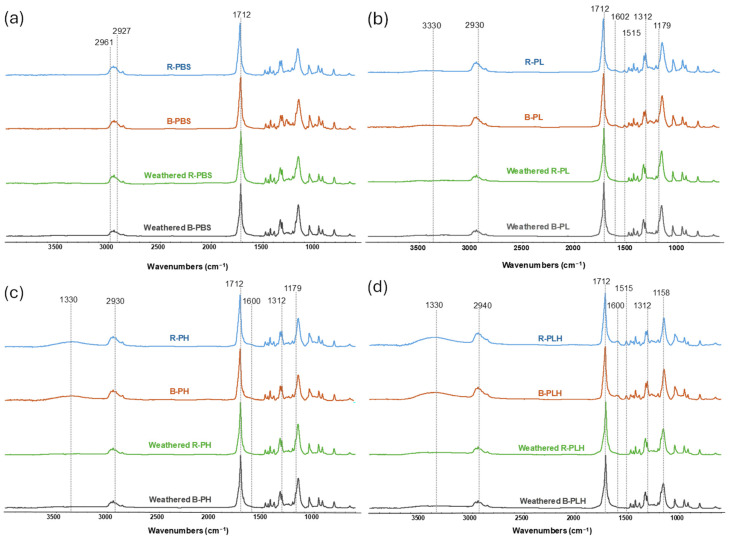
FTIR spectra of control and accelerated weathered (**a**) PBS, (**b**) PBS–lignin, (**c**) PBS–hemp, and (**d**) PBS–lignin–hemp composites.

The surface chemistry of the weathered PBS and PBS-based composites were analyzed by FTIR (Figure 7). Both the B-PBS and R-PBS samples showed a reduction in the C–H stretching shoulders at 2927 and 2961 cm^−1^ as well as a slight carbonyl shift from 1717 cm^−1^ to 1712 cm^−1^ after weathering. Such changes are commonly associated with reactions such as partial degradation of alkyl chains, hydrolysis or UV-induced oxidation, but FTIR alone cannot distinguish the specific reactions involved [53,58].

For the lignin-containing composites (B-PL and R-PL), weathering caused a slight decrease in the OH stretching band (3200–3500 cm^−1^) and a decrease in the lignin-associated bands in the aromatic region (1515 and 1600 cm^−1^); these are consistent with changes in lignin’s aromatic structure under UV exposure [54,56]. The band at 1305 cm^−1^ shifted to 1312 cm^−1^ while the band at 1150 cm^−1^ shifted to 1155 cm^−1^, with a new shoulder forming at 1179 cm^−1^, indicating changes in C–H bending and C–O stretching regions; they do not, on their own, identify specific degradation pathways.

The hemp fiber composites (B-PH and R-PH) showed similar changes, with reductions in the OH band intensity (~3000–3600 cm^−1^) and C–H stretching region (~2800–3000 cm^−1^), suggesting changes in the hydroxyl-rich and aliphatic groups [53]. The hybrid composites (B-PLH and R-PLH) showed the most pronounced changes, showing significant reductions in OH and C–H band intensities, a shift in the carbonyl peak to 1712 cm^−1^, and the disappearance of aromatic bands.

**Figure 8:**
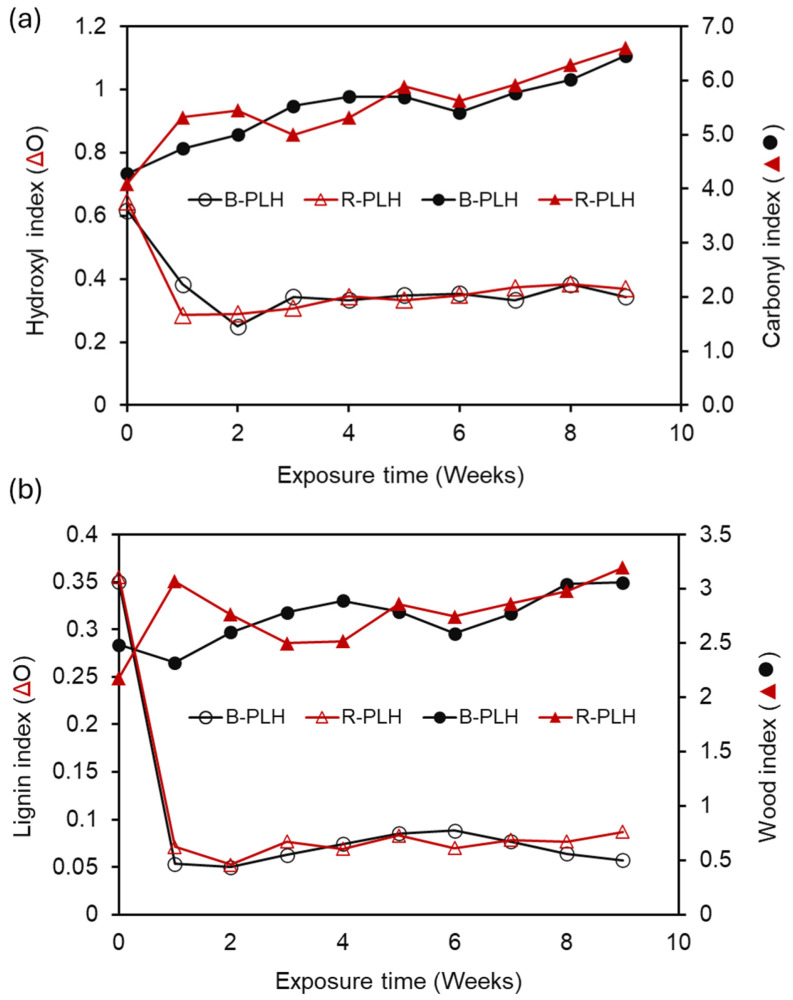
Effect of accelerated weathering on (**a**) hydroxyl (HI) and carbonyl (CI) and (**b**) lignin (LI) and wood (WI) indices of PBS–lignin–hemp composites.

To further study the relative changes in surface chemistry, FTIR indices (HI, CI, LI, WI) were calculated for B-PLH and R-PLH composites (Figure 8).

### 3.10. Fungal Biodegradation of Composites

Both composites showed a decrease in HI, with B-PLH dropping from 0.62 to 0.34 and R-PLH from 0.64 to 0.37 and LI decreasing by over 80% in both formulations. An increase in CIs (B-PLH: 4.28 to 6.46; R-PLH: 4.09 to 6.61) and WIs (B-PLH: 2.48 to 3.06; R-PLH: 2.18 to 3.20) was also observed. These trends suggest relative increases in oxygen-containing groups and the reductions in aromatic contributions, as is consistent with UV-induced modifications. The differences between B-PLH and R-PLH were modest and suggest that DCP’s influence on long-term UV-induced surface modification is limited even if it affects initial composite structure.

Although mechanical tests were not performed post-aging, the observed UV-induced surface oxidation and color change are commonly associated with increased surface brittleness and gradual reduction in flexural and impact properties.

#### 3.10.1. Decay Resistance of PBS–Lignin–Hemp Composites

**Figure 9:**
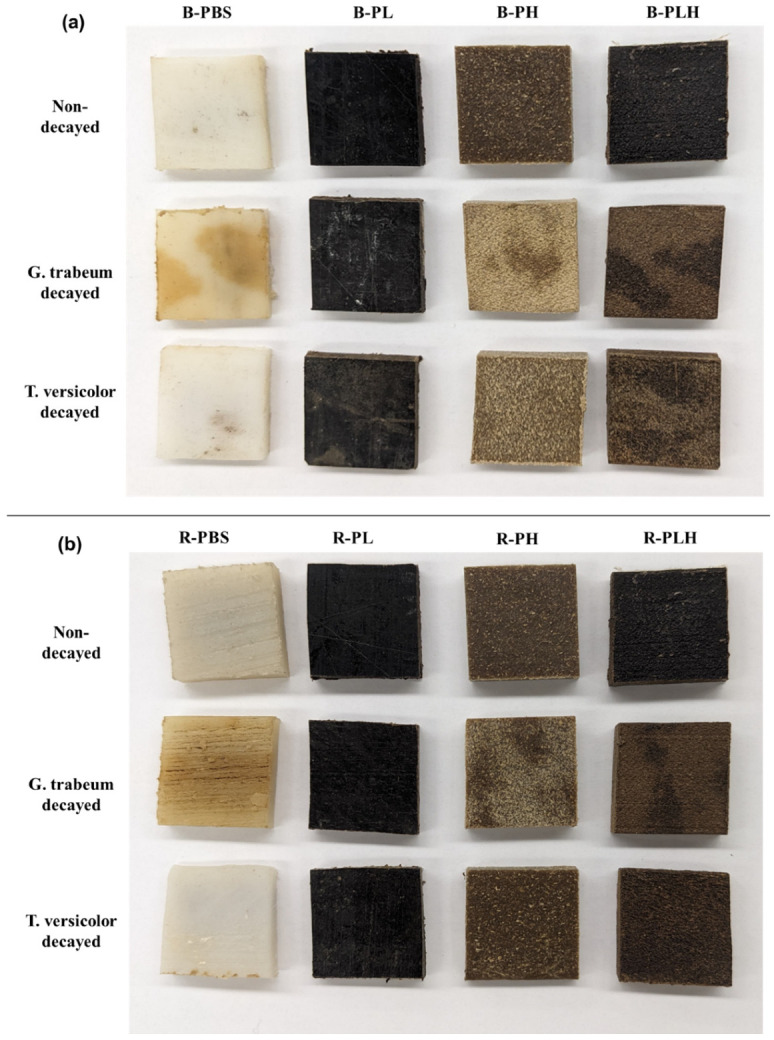
Photographs of original and 16 w fungal degraded (**a**) blended B-PBS, B-PL, B-PH, and B-PLH samples and (**b**) reacted R-PBS, R-PL, R-PH, and R-PLH samples.

Blended and reacted PBS and PBS–lignin–hemp composites were exposed to a brown rot (GT) fungus and a white rot (TV) fungus for 16 weeks, and the photographs of samples before and after fungi exposure are shown in Figure 9. Pine and poplar wood samples exposed to *GT* and *TV*, respectively, after a 4-week exposure period showed moisture content ranging from 60 to 70% and an average mass loss greater than 20%, confirming that the fungal strains used for the test were active. The average mass loss of the test samples after 16 weeks of exposure to *GT* and *TV* is summarized in Table 9. Visual inspection of the composite samples following fungal exposure revealed minimal signs of deterioration, particularly in test samples without hemp fiber inclusion (Figure 9). However, B-PBS and R-PBS samples exposed to *GT* showed noticeable dark-brown discoloration, which may have been masked by the initial dark color of the samples. This discoloration is likely due to oxidative degradation by reactive oxygen species (ROS) produced during fungal metabolism, a known mechanism of brown rot attack [59]. As expected, neat R-PBS samples exposed to *GT* recorded low mass loss (0.11%), given that PBS lacks free biodegradable components. R-PL samples recorded mass loss < 0.3% upon exposure to *GT* fungus (Table 9). This may be due to the inability of the *GT* fungus to degrade lignin, leaving lignin relatively unaltered [60], while DCP improved the density of the composite matrix, restricting fungal hyphae penetration and enzymatic degradation [61,62]. The inclusion of 20% hemp fiber in various composite formulations made the samples more susceptible to fungal decay, with B-PH recording the highest mass loss (1.78%, *p* < 0.05), followed by R-PLH (1.74%), after exposure to fungus *GT*. This increased susceptibility was attributed to the high cellulose and hemicellulose content in hemp, which are readily metabolized by fungi [60]. Correlation analysis shows a strong relationship between water absorption (WA) and mass loss from biodegradation (r = 0.82 for brown rot and *r* = 0.73 for white rot), indicating that higher moisture uptake in test samples increases fungal susceptibility.

A similar trend in results was observed for samples exposed to the white rot fungus *TV*. However, it is worth noting that R-PH samples recorded the highest mass loss (0.57%) upon exposure to *TV*. This can be explained by the 20% biodegradable composition (lignin and hemp fiber) of the crosslinked composite samples evaluated. In comparison, the mass loss of composites (with hemp fiber) exposed to *GT* decay was significantly higher (*p* < 0.05) compared to those exposed to *TV*. Regardless, the relatively low mass loss (<2%) recorded for both B-PLH and R-PLH samples indicates that both composites exhibit fungal resistance and can be considered for use in both indoor and outdoor applications without the need for chemical modifications or treatment procedures. The low fungal mass loss suggests that bulk mechanical integrity would largely be preserved under biological exposure conditions.

#### 3.10.2. SEM Analysis of Decayed Composites

**Figure 10:**
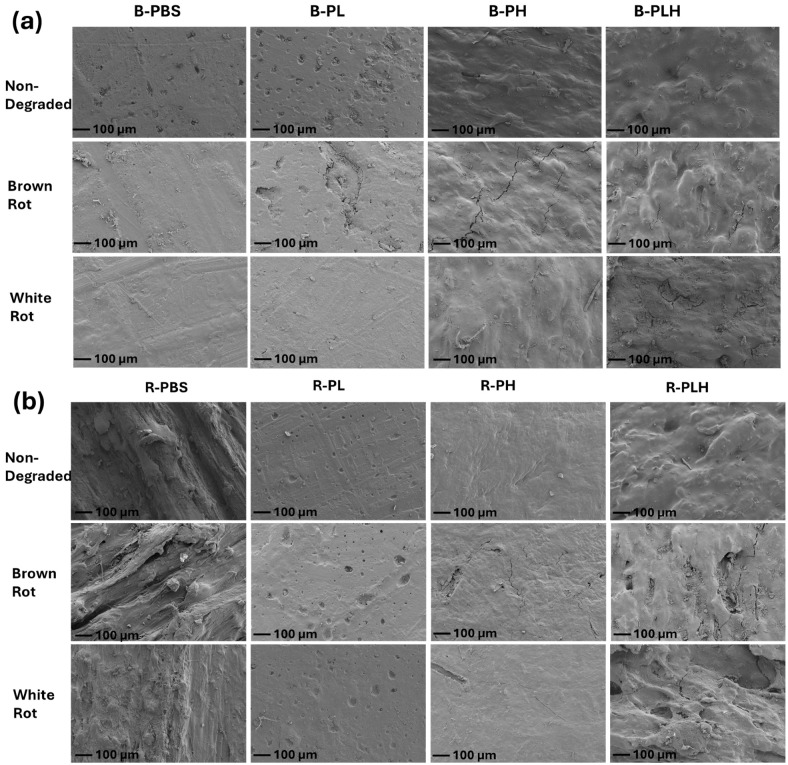
Scanning electron micrographs of original and 16 w fungal degraded (**a**) blended B-PBS, B-PL, B-PH, and B-PLH samples and (**b**) reacted R-PBS, R-PL, R-PH, and R-PLH samples.

The SEM micrographs of the surfaces of PBS and PBS composite after the fungi decay test are shown in Figure 10. The micrograph of B-PBS showed minimal surface damage, indicating the resistance to both brown (*GT*) and white rot (*TV*) fungal attack, as expected. However, R-PBS samples exposed to both fungi were characterized by decay fissures and dents on the surface of the samples. This may be due to the addition of hydroxyl groups into the composite formulation by incorporating DCP, which rendered the samples susceptible to fungal decay. The lignin formulations (B-PL and R-PL) showed minimal surface changes after the fungal exposure. Nonetheless, B-PH and R-PH composite formulations exposed to *GT* were characterized by cracks and fissures along the surface of the samples, while *TV*-exposed samples showed none. This surface analysis corroborates the mass loss data, especially for B-PH (1.78%). The micrographs for B-PLH showed cracks and fissures, which were more pronounced in samples exposed to *TV* than *GT*. However, the introduction of DCP into R-PLH composites showed significant alterations in surface morphology after exposure to both fungi, which were characterized by deep cavities, extensive fissures, and surface erosion. This may be attributed to the increased hydrophilicity and oxidative modifications induced by DCP, which could enhance fungal attachment and enzymatic degradation, leading to more extensive deterioration [49,63].

#### 3.10.3. Surface Chemistry of Biodegraded Composites

**Figure 11:**
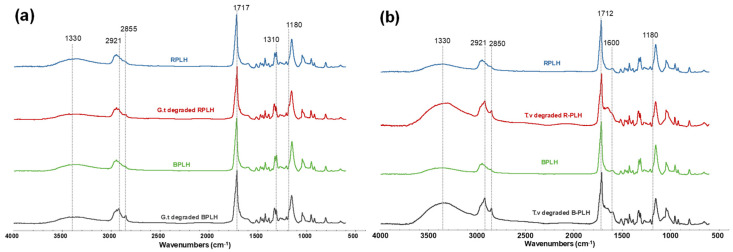
FTIR spectra of non-decayed and (**a**) brown rot and (**b**) white rot fungi-degraded PBS–lignin–hemp composites.

FTIR was employed to analyze the chemical changes in samples after fungal exposure (Figure 11). *GT* degraded B-PLH and R-PLH showed reductions in the O–H stretching band (3000–3600 cm^−1^) and the formation of shoulders at 2855 and 2921 cm^−1^, indicating alterations in hydroxyl- and aliphatic-associated bands. The carbonyl band shifted from 1714 to 1717 cm^−1^, which suggest changes in the chemical environment, like what was observed in UV-weathered samples. The aromatic region (1500–1600 cm^−1^) remained mostly unchanged, suggesting that lignin was comparatively less affected under these conditions. Additionally, a shoulder appeared at 1180 cm^−1^, pointing to modifications in C–O bonds. The general decrease in the band intensity between 950 and 1045 cm^−1^ suggests changes in polysaccharide-related functional groups.

For *TV*-exposed B-PLH and R-PLH, an increase in the O–H stretching band (3000–3600 cm^−1^) was observed, likely due to fungal colonization and the presence of hydroxyl-rich chitin components in fungal cell walls. The C–H stretching region (2948 cm^−1^) shifted to 2921 cm^−1^, and the shoulder at 2850 cm^−1^ became more prominent, suggesting alterations of the aliphatic chains. The C=O stretching peak shifted from 1712 to 1717 cm^−1^, indicating modifications to the ester group. An increase in the absorbance band 1600 cm^−1^, assigned as an amide I of chitin, was observed in *TV*-degraded B-PLH and R-PLH, suggesting fungal hyphae colonization of the samples [63,64]. Additionally, the shoulder at 1180 cm^−1^ intensified, while an increase in intensity from 970 to 1110 cm^−1^ suggests structural changes in lignocellulose components due to fungal metabolism.

**Figure 12:**
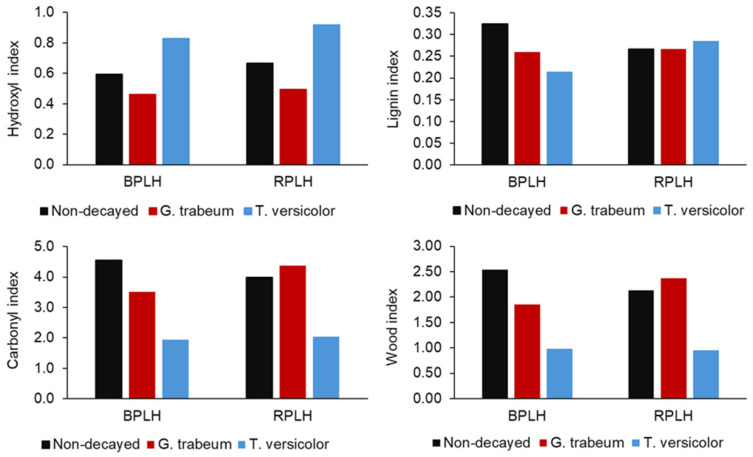
Hydroxyl (HI), lignin (LI), carbonyl (CI), and wood indices (WI) of non-decayed and fungal decayed PBS–lignin–hemp composites.

FTIR indices of decayed samples further clarified these degradation patterns (Figure 12). *GT*-degraded R-PLH showed minimal LI reduction, while B-PLH showed a moderate decrease, consistent with the preserved aromatic bands. *TV* showed contrasting trends. B-PLH’s lignin index decreased, but R-PLH’s increased slightly. This anomaly may reflect competing effects; fungal chitin’s 1600 cm^−1^ amide I band could artificially elevate the lignin index. The WI decreased in *TV*-degraded B-PLH, consistent with fiber breakdown. These indices show that fungal exposure modified oxygen-containing and aromatic groups to different degrees.

## 4. Conclusions

This study evaluated the rheological, mechanical, thermal, weathering, and biodegradation properties of PBS composites reinforced with lignin and milled hemp stalks, with a focus on B-PLH and R-PLH as the final composite products. Rheological analysis showed that lignin reduced viscosity, while hemp fibers increased it significantly, with DCP crosslinking further enhancing viscosity and shear-thinning behavior. Mechanical tests confirmed that lignin and hemp improved flexural strength and stiffness, with B-PLH and R-PLH achieving the highest modulus values, demonstrating positive reinforcement effects. However, tensile strength and elongation at break was lower than neat PBS, reflecting restriction in chain mobility.

Thermal analysis indicated that although lignin and hemp reduced the onset degradation temperature, they significantly decreased mass loss rates and increased char formation, improving overall thermal stability. Water absorption tests showed that hemp increased hydrophilicity, leading to higher swelling, while DCP further increased water uptake in lignin–hemp composites. Accelerated weathering showed that lignin–hemp combinations experienced substantial UV-induced changes, with B-PLH and R-PLH experiencing the most pronounced color and surface changes. Fungal decay tests demonstrated controlled mass loss, with all composites showing ≤2% degradation after extended fungal exposure despite increased moisture uptake. Taken together, durability results show that biological stability and photo-oxidative resistance respond differently to lignocellulosic reinforcement and DCP-assisted reactive extrusion, which emphasizes the need to balance stiffness, durability, and environmental aging resistance when designing PBS-based composites. The results suggest that B-PLH and R-PLH composites balance improved stiffness and thermal stability, making them suitable for rigid, compostable applications. However, their susceptibility to water absorption and UV-induced changes should be considered when designing outdoor applications.

Future studies will focus on systematically optimizing filler ratios and peroxide concentrations to further tailor performance for specific applications.

## Figures and Tables

**Table 1:** Formulation designation and compositions.

Formulation	Composition
B-PBS	PBS (100%)
B-PL	PBS (80%), Lignin (20%)
B-PH	PBS (80%), Hemp Fiber (20%)
B-PLH	PBS (60%), Lignin (20%), Hemp Fiber (20%)
R-PBS	PBS (99.75%), DCP (0.25%)
R-PL	PBS (79.75%), Lignin (20%), DCP (0.25%)
R-PH	PBS (79.75%), Hemp Fiber (20%), DCP (0.25%)
R-PLH	PBS (59.75%), Lignin (20%), Hemp Fiber (20%), DCP (0.25%)

Note: The “B-” prefix denotes simple blended formulations without DCP, while the “R-” prefix signifies formulations prepared through reactive extrusion with DCP. Subscripts indicate the specific material(s) incorporated, such as PL for PBS–lignin, PH for PBS–hemp fiber, and PLH for PBS–lignin–hemp fiber.

**Table 2:** Density, gel fraction, and average particle sizes of PBS–lignin–hemp extruded materials.

Material	Density (g/cm^3^)	Gel Content (%)	Average Particle Size (µm)
Hemp Fiber	1.42		232
Lignin	1.21		166
B-PBS	1.24		
B-PL	1.29		
B-PH	1.31		
B-PLH	1.31		
R-PBS	1.24	93.8	
R-PL	1.28	4.3	
R-PH	1.31	69.6	
R-PLH	1.31	41.2	

**Table 3:** Complex viscosity (η*) of the formulations at 1 Hz and 30 Hz and 140 °C, and the power-law fitted model equations with parameters K and n.

Formulation	η* at 1 Hz (kPa.s)	η* at 30 Hz (kPa.s)	*K* (kPa·s)	*n*	R^2^ Values
B-PBS	2.24	0.452	1.95	0.632	0.950
R-PBS	8.15	0.686	8.22	0.284	0.999
B-PH	9.93	1.54	10.1	0.453	0.997
R-PH	15.7	1.28	15.6	0.274	0.999
B-PL	0.573	0.278	0.516	0.875	0.906
R-PL	0.727	0.279	0.625	0.836	0.896
B-PLH	7.57	1.36	8.52	0.443	0.992
R-PLH	9.41	1.44	10.8	0.382	0.992

**Table 4:** Storage modulus, tan δ, and T_g_ of PBS–lignin–hemp composites.

Formulation	E′ at −48 °C (GPa)	E′ at 10 °C (GPa)	tan δ at −48 °C	tan δ at 10 °C	T_g_ (°C)
B-PBS	0.98	0.28	0.142	0.151	−34
R-PBS	1.14	0.29	0.224	0.139	−33
B-PL	0.81	0.28	0.094	0.122	−27
R-PL	0.85	0.29	0.115	0.120	−26
B-PH	1.01	0.40	0.138	0.117	−33
R-PH	1.21	0.45	0.116	0.109	−29
B-PLH	1.51	0.69	0.114	0.140	−26
R-PLH	1.68	0.81	0.120	0.155	−27

**Table 5:** Melt and crystallization behavior of neat PBS and PBS–lignin–hemp composites determined by DSC.

Formulation	T_m1_ (°C)	T_m2_ (°C)	T_c_ (°C)	ΔH_m_ (J/g)	X_c_ (%)
B-PBS	107	116	84.1	52.4	25.0
R-PBS	-	115	92.7	61.1	29.1
B-PL	101	114	76.8	48.8	29.1
R-PL	102	114	80.0	50.3	29.9
B-PH	105	115	82.3	42.2	25.1
R-PH	-	113	93.9	49.0	29.2
B-PLH	100	112	77.5	36.1	28.6
R-PLH	-	113	74.3	38.5	30.6
100% Crystalline PBS				210	

**Table 6:** Thermogravimetric data for neat PBS, lignin, hemp, and PBS–lignin–hemp composites.

	Major T_onset_ (°C)	DTG_max_ Peak (°C)	Residual at 900 °C (%)	Mass Loss Rate (%/min)
B-PBS	393 ± 0.4	433 ± 2	1.4 ± 0.2	−42
R-PBS	392 ± 0.2	435 ± 2	1.6 ± 0.1	−41
B-PL	385 ± 0.2	418 ± 0.2	10.1 ± 0.6	−32
R-PL	388 ± 1	418 ± 2	10.4 ± 0.4	−35
B-PH	382 ± 3	425 ± 2	7.2 ± 0.4	−31
R-PH	380 ± 1	422 ± 3	6.4 ± 1.1	−32
B-PLH	373 ± 2	418 ± 1	12.0 ± 2.6	−21
R-PLH	370 ± 2	425 ± 2	16.3 ± 0.2	−21
Lignin	320 ± 2	405 ± 0.3	39.3 ± 0.2	−6.4
Hemp Fiber	315 ± 2	353 ± 0.5	20.3 ± 0.4	−18

**Table 7:** Tensile and flexural test values for neat PBS and PBS–lignin–hemp composites. Statistical differences in the results were measured using a pair-wise test (*p*-value < 0.05) and are shown by superscript letters.

Formulation	Tensile Strength (MPa)	Young’s Modulus (MPa)	Flexural Strength (MPa)	Flexural Modulus (MPa)	Elongation at Break (%)
B-PBS	36.7 ± 1.2 ^a^	738 ± 32 ^e^	29.0 ± 2.6 ^d^	701 ± 83 ^g^	15.6 ± 2.8 ^a^
R-PBS	36.4 ± 0.9 ^a^	869 ± 26 ^d^	30.5 ± 0.9 ^cd^	759 ± 49 ^fg^	12.4 ± 3.4 ^ab^
B-PL	28.4 ± 1.1 ^cd^	928 ± 75 ^c^	32.1 ± 1.3 ^c^	830 ± 43 ^ef^	8.3 ± 1.2 ^abc^
R-PL	29.3 ± 1.4 ^c^	967 ± 43 ^c^	35.8 ± 0.9 ^b^	930 ± 20 ^e^	8.2 ± 1.1 ^abc^
B-PH	26.5 ± 2.1 ^d^	1490 ± 126 ^b^	31.8 ± 3.4 ^cd^	1071 ± 108 ^d^	5.6 ± 0.2 ^bc^
R-PH	32.1 ± 0.5 ^b^	1543 ± 37 ^b^	45.5 ± 2.1 ^a^	1370 ± 103 ^c^	5.6 ± 0.3 ^bc^
B-PLH	28.8 ± 1.1 ^cd^	1795 ± 156 ^a^	37.6 ± 0.9 ^b^	1496 ± 133 ^b^	2.4 ± 0.1 ^c^
R-PLH	29.5 ± 2.0 ^c^	1789 ± 85 ^a^	42.6 ± 2.5 ^a^	1785 ± 62 ^a^	2.6 ± 0.2 ^c^

**Table 8:** Water absorption test values for PBS and PBS–lignin–hemp composites. Statistical differences in the results were measured using a pair-wise test (*p*-value < 0.05) and are shown by superscript letters.

Formulation	WA_1 d (%)	WA_56 d (%)	TS_56 d (%)	Diffusion Coefficient (m^2^/s)
B-PBS	0.42 ± 0.1 ^e^	0.95 ± 0.1 ^f^	0.66 ± 0.4 ^c^	6.8 × 10^−12^
R-PBS	0.64 ± 0.0 ^d^	1.08 ± 0.1 ^f^	0.65 ± 0.3 ^c^	8.5 × 10^−12^
B-PL	0.52 ± 0.0 ^de^	2.86 ± 0.2 ^e^	0.67 ± 0.4 ^c^	1.0 × 10^−11^
R-PL	0.53 ± 0.1 ^de^	3.27 ± 0.1 ^d^	0.64 ± 0.3 ^c^	1.1 × 10^−11^
B-PH	1.16 ± 0.1 ^b^	4.35 ± 0.2 ^c^	1.37 ± 0.3 ^bc^	1.2 × 10^−11^
R-PH	0.91 ± 0.0 ^c^	3.60 ± 0.1 ^d^	1.99 ± 0.4 ^b^	1.2 × 10^−11^
B-PLH	1.23 ± 0.1 ^b^	6.88 ± 0.x ^b^	3.49 ± 0.1 ^b^	1.6 × 10^−11^
R-PLH	1.82 ± 0.2 ^a^	8.25 ± 0.4 ^a^	3.44 ± 1.5 ^a^	1.2 × 10^−11^

**Table 9:** Fungal mass loss values for PBS and PBS–lignin–hemp composites.

Fungi	Formulation	Mass Loss (%)
GT	B-PBS	0.11 ± 0.0 ^efg^
TV	B-PBS	0.09 ± 0.0 ^fg^
GT	R-PBS	0.18 ± 0.1 ^efg^
TV	R-PBS	0.22 ± 0.1 ^efg^
GT	B-PL	0.23 ± 0.1 ^ef^
TV	B-PL	0.14 ± 0.1 ^efg^
GT	R-PL	0.17 ± 0.1 ^efg^
TV	R-PL	0.01 ± 0.0 ^g^
GT	B-PH	1.78 ± 0.2 ^a^
TV	B-PH	0.23 ± 0.1 ^ef^
GT	R-PH	0.97 ± 0.0 ^c^
TV	R-PH	−0.04 ± 0.0 ^g^
GT	B-PLH	1.34 ± 0.2 ^b^
TV	B-PLH	0.35 ± 0.1 ^e^
GT	R-PLH	1.74 ± 0.4 ^a^
TV	R-PLH	0.57 ± 0.1 ^d^

Values with the same letter allocation are not statistically different (*p* ≥ 0.05).

## Data Availability

The original contributions presented in this study are included in the article. Further inquiries can be directed to the corresponding author.
